# Epidemiology and Outcome of Acute Appendicitis during and before the COVID-19 Pandemic: A Retrospective Single-Center Analysis

**DOI:** 10.3390/medicina59050902

**Published:** 2023-05-08

**Authors:** Moonho Won, Chiwon Ahn

**Affiliations:** Department of Emergency Medicine, College of Medicine, Chung-Ang University, Seoul 06974, Republic of Korea; wonmh0922@cauhs.or.kr

**Keywords:** COVID-19, appendicitis, emergency department, complication

## Abstract

*Background and Objectives*: We investigated epidemiological factors and outcomes, including the development of complications, for patients with appendicitis according to three sequential coronavirus disease 2019 (COVID-19) pandemic periods, divided by specific time points. *Materials and Methods*: This observational study included patients with acute appendicitis who arrived at a single-center between March 2019 and April 2022. The study divided the pandemic into three periods: period A as the first phase of the pandemic (from 1 March 2020 to 22 August 2021), period B as the time period the medical system stabilized (from 23 August 2021 to 31 December 2021), and period C as the time period of the exploration of patients with COVID-19 in South Korea (from 1 January 2022 to 30 April 2022). Data collection was based on medical records. The primary outcome was presence or absence of complications and the secondary outcomes were the time taken from ED visit to surgical intervention, the presence and time of the first administration of antibiotics, and the hospital stay time. *Results*: Of 1,101 patients, 1,039 were included, with 326 and 711 patients before and during the pandemic, respectively. Incidence of complications was not affected during the pandemic (before the pandemic 58.0%; period A 62.7%; period B,55.4%; and period C 58.1%; *p* = 0.358). Time from symptom onset to emergency department (ED) arrival significantly decreased during the pandemic (before the pandemic 47.8 ± 84.3 h; pandemic 35.0 ± 54 h; *p* = 0.003). Time from ED visit to the operating room was statistically significantly increased during the pandemic (before the pandemic 14.3 ± 21.67 h; period A 18.8 ± 14.02 h; period B 18.8 ± 8.57 h; period C 18.3 ± 12.95 h; *p* = 0.001). Age and time from symptom onset to ED arrival were variables affecting the incidence of complications; however, they were not affected during the pandemic (age, OR 2.382; 95% CI 1.545–3.670; time from symptom onset to ED arrival, OR 1.010, 95% CI 1.006–1.010; *p* < 0.001). *Conclusions*: This study found no differences in postoperative complications or treatment durations between pandemic periods. The incidence of appendicitis complications was significantly influenced by age and the duration between the onset of symptoms and arrival at the emergency department, but not by the pandemic period itself.

## 1. Introduction

In December 2019, coronavirus disease 2019 (COVID-19) emerged in Wuhan, China and soon became a pandemic. During the early stages of the pandemic, the majority of medical resources were focused on the care of patients with COVID-19, and the pandemic impacted the systems of emergency medical centers and affected the diagnosis and treatment processes of patients without COVID-19 [1,2,3]. Furthermore, a gap in emergency medical services developed for other diseases, including myocardial infarction, stroke, out-of-hospital cardiac arrest (OHCA), and sepsis, owing to the collateral effects [2,3,4,5,6,7,8]. Previous studies have revealed that patients with these life-threatening diseases experienced delays in arrival through overburdened emergency medical services and medical institutions with limited capacity, restricting their access to proper care [2,3,4,5,6,7,8]. Additionally, the pandemic’s impact on emergency rooms altered the care provided to patients requiring surgical treatment. Previous studies by Pogoreli et al. discovered a significant increase in delayed presentations and care among patients with testicular torsion during the COVID-19 pandemic [9]. Furthermore, orthopedic surgery, considered a relatively non-life-threatening condition, experienced delays in stages to alleviate the strain on healthcare services [10].

Acute appendicitis is one of the common causes of acute abdomen and is the most common indicator for emergency abdominal surgery. Previous studies have reported that during the pandemic, a significant increase in complications and length of stay for acute appendicitis was noted, compared with that from before the COVID-19 pandemic [11,12,13]. Köhler et al. reported that during the pandemic, adult and child patients with appendicitis were receiving longer wait times, and their postoperative complications were worsened [11]. Furthermore, they showed that the time it took from the onset of symptoms to arriving at the emergency department (ED) increased during the pandemic; however, it was not statistically significant [11]. Access to EDs was restricted compared to before the pandemic, and patients had a generalized fear of infection risk from the novel virus [14,15]. Moreover, additional procedures during diagnostic testing and subsequent surgery, including the need for personal protection of the clinician and care in an isolation area when the patient presents symptoms and signs of a febrile illness, and the proactive administration of a COVID-19 polymerase chain reaction (PCR) test preoperatively, may lengthen the time until the surgery [2].

As the pandemic continued, the medical system stabilized, in contrast to the earlier temporally collapsed environment. At this time, distancing measures and restrictions on social activity were loosened, medicines for COVID-19 were developed, vaccines were administered, and a subtype with a high rate of transmission and a low rate of severity became the predominant type. Owing to the altered treatment environment, generalizing earlier outcomes for appendicitis during the pandemic is difficult. For this reason, it is necessary to divide the pandemic period into early and late phases.

Unlike previous studies, we investigated epidemiological factors and outcomes, including the development of complications for patients with appendicitis, according to three sequential pandemic periods divided by specific time points.

## 2. Materials and Methods

### 2.1. Study Design and Population

This study was a single-institutional retrospective observational study that included all ages with acute appendicitis who arrived at our ED between 1 March 2019 and 30 April 2022. Reviewing data were obtained from the electronic medical records and were analyzed. The 30-bed center has an ED staffed by board-certified emergency physicians providing 24-h emergency service. Among them, those who were transferred to other hospitals following diagnosis, those who did not proceed with the final treatment at the hospital owing to treatment refusal, those who were finally excluded from diagnosis, and those who had insufficient relevant data in their medical records were excluded from this study.

### 2.2. During and before the Pandemic Period, and the Changed Environment in the Emergency Room during the Pandemic

Following the deceleration of the pandemic announced by the World Health Organization in March 2020, we established the classification of a period in the COVID-19 pandemic (from 1 March 2020 to 30 April 2022) and before the pandemic (from 1 March 2019 to 28 February 2020).

The COVID-19 pandemic period was sequentially divided by several time points. Time points included the time when the first vaccination rate reached 51% in South Korea (23 August 2021) and the time when the number of confirmed cases, following the Omicron epidemic, exploded (January 2022). Thus, the pandemic period was divided into the following three periods: period A as the first phase of the pandemic (from 1 March 2020 to 22 August 2021), period B as the time period the medical system stabilized (from 23 August 2021 to 31 December 2021), and period C as the time period of the exploration of patients with COVID-19 in South Korea (from 1 January 2022 to 30 April 2022).

The emergency medical staff and the infection control team of this institution established and modified the ED clinical processes to prevent infection spread in the ED during the COVID-19 pandemic, in accordance with the government of South Korea’s elevation of the infectious disease crisis level to “serious” in February 2020. Due to the COVID-19 pandemic and the implementation of a modified clinical process, the triage area was established in a separate location outside the ED [2]. After screening, patients with COVID-19-related symptoms or clinical suspicion of COVID-19 were led to the isolation room with negative pressure, instead of the space for patients with other general symptoms. Staff, residents, nurses, and healthcare assistants wore personal protective equipment consisting of a protective gown, a protective cap, gloves, a N95 mask, and a face shield or goggles [16]. COVID-19 reverse transcription-polymerase chain reaction testing was performed on all patients residing in the isolated room. If the decision to hospitalize was made prior to the results of a confirmatory COVID-19 test, the patients were transferred to the isolation intensive care unit or isolation wards via a different route.

### 2.3. Data Collection and Outcome Measurement

Data collection is based on the electronic medical records of the study participants and includes basic patient information (e.g., age, gender, and pregnancy), patient evaluation information (e.g., hypertension, diabetes mellitus, cancer, stroke, cardiovascular disease, and surgical history), and acute appendicitis-related information (e.g., time from symptom onset to ED visit, time from ED visit to surgical intervention, reading of ultrasonography or computed tomography by a radiologist, and hospital stay time).

The presence or absence of complications was the primary outcome; the time taken from ED visit to surgical intervention, the presence and time of the first administration of antibiotics, and the hospital stay time were secondary outcomes.

### 2.4. Statistical Analysis

Descriptive statistical analysis was applied to ascertain baseline characteristics. Categorical variables were expressed as frequencies and were analyzed using the Chi-square test or Fisher’s exact test. Continuous variables were expressed as means with standard deviations and were analyzed using Student’s T-test. The Kolmogorov–Smirnov test was used to test for the normality of data distribution in all datasets. To identify predictors of outcomes, intergroup covariates, including the binary variable of complication development, were evaluated using multivariate analysis, which was independently performed by logistic regression using the “enter” method. Age (e.g., child, adult, and older adult), surgical history, time from onset, and time to antibiotics from onset were adjusted.

We used the Jamovi statistical program (version 2.3.18, The Jamovi project, Sydney, Australia) and R program (version 4.2.2, The R Foundation for Statistical Computing, Vienna, Austria) for all statistical analyses. A *p*-value of <0.05 was considered statistically significant.

### 2.5. Ethics Statement

This study’s protocol was approved by the Institutional Review Board of the Chung-Ang University Hospital on 24 August 2022 (IRB No. 2206-021-19424). The requirement for informed consent was waived owing to the retrospective nature of the study.

## 3. Results

### 3.1. Population and Demographics

**Figure 1:**
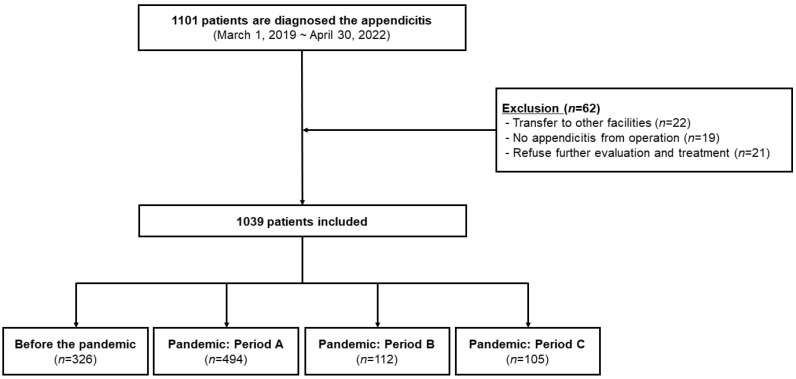
Flow chart of included patients.

A total of 1101 patients were included in this study, and 62 patients were excluded owing to various reasons, including transfer to other institutions, no appendicitis from the final diagnosis, and refusal for further evaluation and treatment. Finally, 1039 patients were analyzed, of whom 326 and 711 patients were reported before and during the pandemic period, respectively (period A, 494 patients; period B, 112 patients; and period C, 105 patients) (Figure 1).

Comparing between before and during the pandemic period, no statistically significant difference was observed in the rates of gender and underlying diseases. The time from symptom onset to ED visit significantly decreased (before the pandemic, 47.8 ± 84.3 h; during the pandemic, 35.0 ± 54 h; *p* = 0.003) (Table 1).

### 3.2. Outcomes

The ratio of appendicitis to the total patients in each period was 0.76% before the pandemic period (326/42,857 patients), 1.11% in period A of the pandemic (494/44,403 patients), 0.85% in period B of the pandemic (112/13,185), and 1.03% in period C of the pandemic (105/10,107 patients).

**Figure 2:**
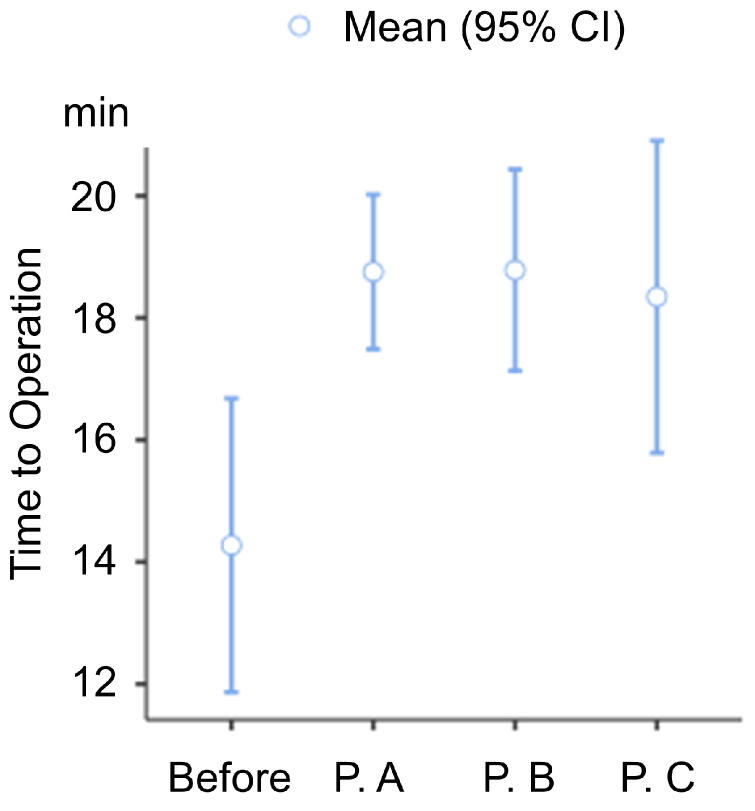
Box plot for time to operation according to each period, including the pandemic and before the pandemic. **P.A**, Period A; **P.B**, Period B; **P.C**, Period C.

The incidence of complications was not affected during the pandemic period (before the pandemic, 58.0%; period A, 62.7%; period B, 55.4%; and period C, 58.1%; *p* = 0.358). The time from ED to the operating room was statistically significantly increased (before the pandemic, 14.3 ± 21.67 h; period A, 18.8 ± 14.02 h; period B, 18.8 ± 8.57 h; and period C, 18.3 ± 12.95 h; *p* = 0.001) (Figure 2).

No statistically significant difference was noted in the time from symptom onset to ED admission, time from symptom onset to antibiotic administration, hospital stay, and antibiotic treatment only without surgical treatment (Table 2).

### 3.3. Influencing Factors for Incidence of Complications

As a result of logistic regression analysis by adjusting for age, surgical history, time from symptom onset to ED, and time from ED to antibiotic administration, old age (odds ratio [OR], 2.382; 95% confidence interval [CI], 1.545–3.670; *p* < 0.001) and time from symptom onset to ED (OR, 1.010; 95% CI, 1.006–1.010; *p* < 0.001) were shown to be variables affecting the incidence of complications (Table 3).

## 4. Discussion

This study, unlike previous studies, investigated complications and other factors for appendicitis throughout the pandemic period by dividing it into multiple time points and comparing it to the period before the pandemic. The occurrence of complications was influenced by old age and the duration between symptom onset and ED arrival; however, the period of pandemic had no effect. During the pandemic, the duration between ED and surgical treatment dramatically increased; however, the time between symptom onset and ED arrival significantly decreased. Contrary to the study’s hypothesis, each factor throughout the divided pandemic periods lacked a significant difference.

COVID-19 is a new infectious disease, and the fear of spread, lack of vaccines and treatments, and confusion in the medical system owing to the absence of treatment guidelines affected the treatment process for both infectious and non-infectious diseases in the early stages of the pandemic. As the pandemic progressed, patients who had signs or symptoms, including fever, or were suspected of having the infectious disease, were admitted to an isolation room for treatment [2,17,18,19]. Additionally, although patients did not have the infectious disease or were not suspected of having it, they were required to undergo the routine COVID-19 PCR test for hospital admission and surgery [20,21,22,23]. Moreover, surgical times were delayed owing to pending COVID-19 PCR test results, which were confirmed only after the surgery’s schedule had been determined. Even when appendicitis was diagnosed in the ED, these additional processes led to the delay in reaching the operating room. In a study by Kim et al., which was a single-center study conducted in Korea during the pandemic, surgeries were delayed, and the severity of inflammation increased as a result of the delays caused by extra processes preoperatively [24]. Since it is not only the surgical delay that affects the occurrence of complications but also the duration of antibiotic administration, old age, and the presence of comorbidities [25,26,27,28], it appears that the increase in the time to start surgery during the pandemic had no direct effect on the increase in complications in this study.

The development of a drug for COVID-19 infection and the widespread use of vaccines both contributed to decreasing the number of patients with COVID-19 who were in a serious condition and altered the dominant subtype of the virus [29]. Omicron, in particular, had a low severity rate but a rapid spreading rate [30,31]. Despite these, incidents have changed the environment of medical care. Distinguishing between patients with and without COVID-19 to prevent the spread of the virus and maintaining strict infection control measures to protect vulnerable patients remain necessary. However, despite dividing the pandemic into periods for study, we were unable to figure out the difference from each period.

In previous studies, only antibiotic treatment without surgery was observed to have significantly increased during the pandemic compared with that before the pandemic [11]. Owing to the limited surgical capacity available during the pandemic period [11], antibiotic treatment without surgery increased since it could be a suitable choice for treating uncomplicated appendicitis [32,33]. All patients in this study received antibiotics; however, the number of patients who did not undergo surgery did not significantly increase during the pandemic. Although it can be presumed that the altering medical environment brought on by the pandemic had no impact on a surgical decision, a single-center study may call for more investigations into how various institutions in South Korea changed their surgical environments owing to the pandemic.

Compared with that before the pandemic, the amount of time between symptom onset and ED arrival significantly decreased. No studies showed a reduction in the amount of time between symptom onset and ED arrival in patients during the pandemic. There is evidence, however, that the onset of the pandemic has resulted in a novel infectious disease that has never been encountered, which has contributed to a decrease in the overall number of patients visiting hospitals owing to reduced social activities, including social distancing and self-isolation, as well as reduced access to hospitals for mildly ill patients [34]. It is probable that this is the collateral effect of the pandemic, which has led to a decrease in overall patient visits to the ED and an increase in accessibility for patients with urgent conditions, such as appendicitis.

Although this study did not find a significant difference in the incidence of complications between children and adults, delayed diagnosis of acute appendicitis in children [35,36,37] can make the condition more severe. An earlier systematic review of the incidence of complications in children with pandemics revealed that the incidence of appendicitis with increased complications was high in children with pandemics [38]. Additionally, the frequency of non-surgical treatments increased during the pandemic, contrary to the findings of this study. This is likely the result of regional differences in pandemic-affected health care systems and institutional care process characteristics. Since this study relied on the experience of a single institution, it will be necessary in the future to analyze its characteristics by collecting and analyzing data from multiple nearby institutions.

This study had several limitations. First, this study was conducted at a single center. The results of the study could not be generalized because only local features were considered, it was not possible to compare the treatment protocols of different hospitals, and only one center’s protocol was investigated. Second, the timing of major COVID-19-related health policy events was used to divide the study period; nevertheless, following the protocol at our facility, there was little change in the course of therapy. A considerable difference in each feature may have been shown if the treatment method had clearly changed over time; however, the fact that this was not achieved is a limitation. Finally, this was a retrospective study; therefore, it was impossible to completely rule out the inclusion of selection bias and other uncontrollable variables.

## 5. Conclusions

We hypothesized that the pandemic might cause a delay in appendicitis treatment, and we divided the pandemic into the early period, the medical system stabilization period, and the COVID-19 spread period in South Korea to determine the impact of characteristics and outcomes. There was no difference in postoperative complications among patients with appendicitis at each stage of analysis. In addition, there was no difference between the time periods for surgery after symptom onset and antibiotic administration. The incidence of appendicitis complications was significantly influenced by age and the lengthening of the time between symptom onset and ED arrival, although not by the pandemic period. Additionally, compared to before the pandemic, the time from the ED to surgical intervention increased during the pandemic period owing to additional processes, including COVID-19 screening and care in an isolation state if the patient had signs or symptoms of infection.

## Figures and Tables

**Table 1:** Baseline characteristics.

	Before the Pandemic (*n* = 326)	Pandemic (*n* = 711)	*p*-Value
Sex, male	148 (45.4%)	343 (48.2%)	0.395
Age, years	39.0 ± 20.6	42.4 ± 20.4	0.012 0.009
Child (<15)	44 (13.5%)	57 (8.0%)	
Adult (15–64)	242 (74.2%)	537 (75.5%)	
Eldely (≥65)	40 (12.3%)	117 (16.5%)	
Underlying disease			
Hypertension	39 (12.0%)	113 (15.9%)	0.097
Diabetes mellitus	18 (5.5%)	40 (5.6%)	0.946
Cancer	11 (3.4%)	27 (3.8%)	0.736
Stroke	3 (0.9%)	12 (1.7%)	0.337
Cardiovascular disease	11 (3.4%)	25 (3.5%)	0.908
History of operation	133 (40.8%)	241 (33.9%)	0.032
Time from symptom onset to ED, min	47.8 ± 84.3	35.0 ± 54.1	0.003

ED, emergency department.

**Table 2:** Clinical characteristics and outcomes according to pandemic period.

Variables	Before the Pandemic (*n* = 326)	Pandemic				*p*
		Period A (*n* = 494)	Period B (*n* = 112)	Period C (*n* = 105)	Total	
Symptom onset, hour	47.8 ± 84.3	35.3 ± 51.4	39.0 ± 61.8	29.7 ± 57.6	35.0 ± 54.1	0.054
Time from ED to antibiotics injection, min	50.9 ± 84.3	38.4 ± 51.4	42.5 ± 62.0	32.7 ± 57.4	38.2 ± 54.1	0.053
Time from ED to operation room, hour	14.3 ± 21.67	18.8 ± 14.02	18.8 ± 8.57	18.3 ± 12.95	18.7 ± 13.1	0.001
Hospital stay, day	4.59 ± 2.88	4.84 ± 2.50	4.72 ± 2.51	4.51 ± 1.78	4.78 ± 2.41	0.451
Complication	189 (58.0%)	311 (62.7%)	62 (55.4%)	61 (58.1%)	434 (60.1%)	0.358
Peritonitis	53 (16.3%)	86 (17.3%)	22 (19.6%)	36 (34.3%)	144 (20.2%)	
Perforation	100 (30.7%)	172 (34.7%)	29 (25.9%)	15 (14.3%)	216 (30.3%)	
Abscess	36 (11.0%)	53 (10.7%)	11 (9.8%)	10 (9.5%)	74 (10.4%)	
Percentage without surgery	12 (3.7%)	21 (4.3%)	6 (5.4%)	4 (3.8%)	31 (4.4%)	0.738

ED, emergency department

**Table 3:** Multivariable logistic regression analysis of complication development of acute appendices by pandemic group.

	B	S.E.	OR	95% CI	*p*
Intercept	0.1025	0.24869	0.773	0.559–1.07	0.121
Sex	0.23265	0.13188	1.262	0.974–1.63	0.078
History of operation	0.08886	0.14318	0.915	0.691–1.21	0.535
Time from symptom onset to ED	0.00961	0.00961	1.010	1.006–1.01	**<0.001**
Age					
Adult	(ref) *				
Child	0.10651	0.22161	1.112	0.720–1.72	0.631
Elderly	0.86796	0.22077	2.382	1.545–3.67	**<0.001**
Period					
Before the pandemic	(ref) **				
Pandemic: Period A	0.26458	0.15201	1.303	0.967–1.76	
Pandemic: Period B	−0.02126	0.22918	0.979	0.625–1.53	
Pandemic: Period C	0.10545	0.24024	1.111	0.694–1.78	

Nagelkerke R^2^ = 0.0904. The data are given as the value; *p* < 0.05 value was accepted as significant level and shown in bold. The *p*-value is the result of using logistic regression (Age (Child, Adult, Elderly), history of operation, time from onset, and time to antibiotics from onset were adjusted). ED, emergency department. * Each group was compared with the adult group as the reference. ** Each group was compared with the before the pandemic group as the reference.

## Data Availability

The datasets generated during the current study are available from the corresponding author on reasonable request.
